# Partitioning seasonal stem carbon dioxide efflux into stem respiration, bark photosynthesis, and transport-related flux in Scots pine

**DOI:** 10.1093/jxb/erae242

**Published:** 2024-05-23

**Authors:** Paulina Dukat, Teemu Hölttä, Ram Oren, Yann Salmon, Marek Urbaniak, Timo Vesala, Juho Aalto, Anna Lintunen

**Affiliations:** Institute for Atmospheric and Earth System Research/Forest Sciences, Faculty of Agriculture and Forestry, University of Helsinki, Helsinki, Finland; Laboratory of Meteorology, Department of Construction and Geoengineering, Faculty of Environmental Engineering and Mechanical Engineering, Poznan University of Life Sciences, Piątkowska 94, 60-649 Poznań, Poland; Institute for Atmospheric and Earth System Research/Forest Sciences, Faculty of Agriculture and Forestry, University of Helsinki, Helsinki, Finland; Institute for Atmospheric and Earth System Research/Forest Sciences, Faculty of Agriculture and Forestry, University of Helsinki, Helsinki, Finland; Nicholas School of the Environment & Pratt School of Engineering, Duke University, Durham NC, USA; Institute for Atmospheric and Earth System Research/Forest Sciences, Faculty of Agriculture and Forestry, University of Helsinki, Helsinki, Finland; Institute for Atmospheric and Earth System Research/Physics, Faculty of Science, University of Helsinki, Helsinki, Finland; Laboratory of Meteorology, Department of Construction and Geoengineering, Faculty of Environmental Engineering and Mechanical Engineering, Poznan University of Life Sciences, Piątkowska 94, 60-649 Poznań, Poland; Institute for Atmospheric and Earth System Research/Forest Sciences, Faculty of Agriculture and Forestry, University of Helsinki, Helsinki, Finland; Institute for Atmospheric and Earth System Research/Physics, Faculty of Science, University of Helsinki, Helsinki, Finland; Institute for Atmospheric and Earth System Research/Forest Sciences, Faculty of Agriculture and Forestry, University of Helsinki, Helsinki, Finland; Institute for Atmospheric and Earth System Research/Forest Sciences, Faculty of Agriculture and Forestry, University of Helsinki, Helsinki, Finland; Institute for Atmospheric and Earth System Research/Physics, Faculty of Science, University of Helsinki, Helsinki, Finland; Lawrence Berkeley National Laboratory, USA

**Keywords:** CO_2_ efflux, CO_2_ transport in xylem sap, sap flow, Scots pine, stem photosynthesis, stem respiration

## Abstract

Stem CO_2_ efflux is an important component of the carbon balance in forests. The efflux is considered to principally reflect the net result of two dominating and opposing processes: stem respiration and stem photosynthesis. In addition, transport of CO_2_ in xylem sap is thought to play an appreciable role in affecting the net flux. This work presents an approach to partition stem CO_2_ efflux among these processes using sap-flux data and CO_2_-exchange measurements from dark and transparent chambers placed on mature Scots pine (*Pinus sylvestris*) trees. Seasonal changes and monthly parameters describing the studied processes were determined. Respiration contributed most to stem net CO_2_ flux, reaching up to 79% (considering the sum of the absolute values of stem respiration, stem photosynthesis, and flux from CO_2_ transported in xylem sap to be 100%) in June, when stem growth was greatest. The contribution of photosynthesis accounted for up to 13% of the stem net CO_2_ flux, increasing over the monitoring period. CO_2_ transported axially with sap flow decreased towards the end of the growing season. At a reference temperature, respiration decreased starting around midsummer, while its temperature sensitivity increased during the summer. A decline was observed for photosynthetic quantum yield around midsummer together with a decrease in light-saturation point. The proposed approach facilitates modeling net stem CO_2_ flux at a range of time scales.

## Teaser

Using continous measurements, net stem CO_2_ flux in Scots pine was partitioned into respiration of up to 80%, photosynthesis of up to 13%, and CO_2_ transported axially with sap flow.

## Introduction

Stem CO_2_ efflux is an important component of the forest carbon balance (Bužková *et al.*, 2015), representing the net result of two dominating processes: stem respiration and stem photosynthesis. Stem respiration has been the subject of many studies, including some in the boreal region (Zha *et al.*, 2004; Hölttä and Kolari, 2009; Kolari *et al.*, 2009). It can account for a large share of total ecosystem respiration (Ilvesniemi *et al.*, 2009). Average stem respiration rate of boreal Scots pine (*Pinus sylvestris*) forest was estimated as 62 g C m^−2^ a^−1^, or ~8% of annual ecosystem respiration obtained with eddy covariance (826 g C m^−2^ a^−1^), and more than 20% of annual canopy respiration (316 g C m^−2^ a^−1^; Ilvesniemi *et al.*, 2009). Even higher values were recorded in ecosystems at lower latitudes. At a temperate mixed forest composed of *Quercus alba*, *Quercus prinus*, and *Acer rubrum*, respiration of stems and branches ranged from 149 to 204 g C m^−2^ a^−1^ (Edwards and Hanson, 1996), exceeding deciduous leaf respiration of mature yellow poplar forest (*Liriodendorn tulipifera*) by more than 2.5-fold (Edwards *et al.*, 1981; Edwards and Hanson, 1996). Farther south in tropical forests, the same components were estimated to account for a quarter of total ecosystem respiration (Chambers *et al.*, 2004; Cavaleri *et al.*, 2006; Rowland *et al.*, 2018).

The second primary process affecting net stem CO_2_ flux is photosynthetic refixation (Osmond *et al.*, 1987; Comstock *et al.*, 1988; Sprugel and Benecke, 1991; Nilsen, 1995; Cernusak and Marshall, 2000; Tarvainen *et al.*, 2018). Most stems of woody plants have chlorophyll-containing tissues, using stem-internal CO_2_ and light penetrating the bark to photosynthesize (Pfanz *et al.*, 2002). Unlike leaves, stems are not specialized for photosynthesis (Berveiller *et al.*, 2007), and thus typically the rate of bark photosynthesis is much lower (Aschan *et al.*, 2001; Berveiller *et al.*, 2007). Accordingly, stem photosynthesis does not result in net carbon gain on a whole-tissue level, but reduces the efflux of CO_2_ from the stem to atmosphere by refixing some of the CO_2_ produced in respiration (Sprugel and Benecke, 1991). Nonetheless, stem photosynthesis plays an important function in tree carbon balance, especially in trees with green stems (Kocurek *et al.*, 2020) and during the leafless periods or periods when foliar photosynthesis is limited by drought stress (Bloemen *et al.*, 2016*b*). Plants with photosynthesizing stems gain additional carbon that may partially compensate for lower foliage carbon gain during drought (Ávila-Lovera *et al.*, 2017), potentially playing a role in drought tolerance (Cernusak and Cheesman, 2015). Despite its potential importance, stem photosynthesis is often neglected, especially in trees where the green bark cells are hidden under a layer of bark (Yiotis *et al.*, 2009). The seasonal and diurnal variability of stem photosynthesis is not well known, because most studies have focused on campaigns generating sampling points in time; here we report results from continuous flux measurements over the 4 month growing season.

CO_2_ originating from soil CO_2_ production and dissolved in water taken up by roots can be transported upward with the transpiration flux, especially during times of high sap flow rates (Bloemen *et al.*, 2014). In addition, a significant amount of the CO_2_ respired in living root and stem cells can dissolve in xylem sap and be transported upwards (axially) in the stem (McGuire *et al.*, 2007), thus affecting the net stem CO_2_ flux (Teskey and McGuire, 2002). Advection, the transport of CO_2_ by the xylem sap, can occur in the presence of an axial CO_2_ concentration gradient (e.g. Teskey *et al.*, 2008; Vesala *et al.*, 2008) arising from the respired carbon dissolved as CO_2_ in the sap. Dissolved carbon continues moving upward with the sap, escaping by diffusion to the atmosphere higher up (McGuire and Teskey, 2004; Acosta *et al.*, 2011; Bužková *et al.*, 2015), assimilating in photosynthesis in stem or leaves (Cernusak and Marshall, 2000; Hibberd and Quick, 2002; Bloemen *et al.*, 2013), or reaching the leaves and diffusing to the atmosphere (Levy *et al.*, 1999). It is not yet fully known how CO_2_ transport in the xylem sap is influenced by the increasing sap-flow rate, as previous studies present different relationships between stem CO_2_ efflux and sap flow rate (Levy *et al.*, 1999; Bowman *et al.*, 2005; Cerasoli *et al.*, 2009; Bužková *et al.*, 2015). For example, Bužková *et al.* (2015) found that stem CO_2_ efflux and sap flow were positively correlated during optimal soil moisture conditions, while they were not correlated during dry conditions. Increasing sap flow rate has also been related to a decrease in local absolute stem CO_2_ efflux (Gansert and Burgdorf, 2005; McGuire *et al.*, 2007; Acosta *et al.*, 2008), presumably because more CO_2_ respired at the measured position was moving up with the sap lowering the amount of CO_2_ prone to radial diffusion out of the stem. However, other studies found that only a small amount of CO_2_ is transported up the stem during summer because ~97% of locally respired CO_2_ can diffuse radially to the atmosphere (Tarvainen *et al.*, 2021). Indeed, sap flow may have a different effect on the transport of CO_2_ depending on the axial CO_2_ concentration gradient in the stem. It should be noted that the three main processes—respiration, photosynthesis, and axial CO_2_ transport in the sap—occur over various intervals of stem depth and the integration of the three fluxes is measured as net flux. Moreover, spatial patterns in the efflux of CO_2_ transported in xylem sap may be influenced by wood anatomy and branch distribution (Salomón *et al.*, 2021).

There are additional processes affecting stem carbon dynamics and potentially contributing to reduction of daytime stem CO_2_ efflux. We aggregate these processes together with the transport term, which in this study includes the residual of parameterized respiration and measured dark-chamber efflux and the uncertainty of these process estimates. These additional processes include non-photosynthetic phosphoenolpyruvate (PEP) carboxylase-mediated fixation (Hilman *et al.*, 2019; Helm *et al.*, 2023), increase in the rate of axial diffusion of respired CO_2_ (Saveyn *et al.*, 2008; De Roo *et al.*, 2019, 2020a), diurnal changes in cell growth rates and related mitochondrial respiration (Hsiao, 1973; Landsberg, 1986; Saveyn *et al.*, 2008; Salomón *et al.*, 2018; Zweifel *et al.*, 2021), and changes in the stem CO_2_ storage. Expecting the physical advective axial transport term to dominate over these additional processes, we hereafter, refer to the three major processes contributing to stem efflux: respiration, stem photosynthesis, and transport.

The processes responsible for the stem CO_2_ efflux are each driven by different variables. Stem respiration includes growth respiration and maintenance respiration (McCree, 1970; Rowland *et al.*, 2018), the latter depending strongly on stem temperature (Amthor, 1989; Ciais *et al.*, 2005; Saveyn *et al.*, 2008). A study on ponderosa pine (*Pinus ponderosa*) in different environments (desert and montane) showed that the response of stem respiration for a 10 °C increase in temperature (*Q*_10_) varied seasonally (Carey *et al.*, 1997); *Q*_10_ generally increased with decreasing prevailing temperatures, being highest in winter. Similarly, *Picea abies* had higher *Q*_10_ in early summer, decreasing towards October (Acosta *et al.*, 2008). In contrast, the main determining factor for stem photosynthesis is light intensity. After passing through the outermost bark layers, the light energy in the range of photosynthetically active radiation (PAR) is utilized by the green tissue of the stem (Kocurek *et al.*, 2020). Like in leaves, stem photosynthesis also depends on temperature, a driver of most physiological processes (Kramer and Kozlowski, 1979; Wittmann and Pfanz, 2007). Seasonal changes in stem photosynthesis were previously found to be driven by temperature and vapor pressure deficit (Nilsen and Sharifi, 1994). In the same study, there were no major differences in quantum yield or light compensation point of the stem photosynthetic light-response curves between spring and summer. Nevertheless, light-saturation point at ambient temperature decreased during the season for one species (*Chloris virgata*). Still, respiration and photosynthesis have different responses to temperature; the rate of respiration is more sensitive to temperature over the range prevailing during the growing season (Maier *et al.*, 1998; Wittmann and Pfanz, 2007). The axial transport of respired CO_2_ in xylem sap is advective and in general depends on the sap flow rate and the vertical gradient of CO_2_ concentration in the sap; the partial pressure of CO_2_ is high in the air space of soil due to microbial respiration, and in wood due to root and stem respiration, while intercellular partial pressure of CO_2_ in leaves is lower (Levy *et al.*, 1999). The dependence of net stem CO_2_ efflux on sap flow daily and seasonally is generated by the axial CO_2_ concentration gradient in the stem. Beside respiration, photosynthesis, and transport themselves, the amount of dissolved CO_2_ in the sap depends also on temperature via Henry’s law and pH via CO_2_ dissociation in the water (Teskey *et al.*, 2008). Temperature-dependent changes explained about half of the seasonal and diurnal variations in xylem CO_2_ concentration in the lower stem of *P. abies* (Etzold *et al.*, 2013; Tarvainen *et al.*, 2014). Tarvainen *et al.* (2021) estimated the role of the different stem CO_2_ efflux components in *P. sylvestris* using ^13^C-labelling. They found that a large fraction of the local respiration diffused radially out of the stem, accounting for most of the measured net CO_2_ efflux. Respiration represented on average 95% of the observed CO_2_ efflux, and photosynthetic refixation was the second most important component of the net CO_2_ flux, at maximum 10% of respiration. The ratio between transport and respiration did not exceed 6%.

In this study, we focus on the two main processes—respiration and photosynthesis—assigning the residual as a transport term, which we are able to explain with sap flow rate. We relied on continuous measurements to quantify the response dynamics of each process during the most active period of the growing season, reflecting seasonal pattern in sensitivities to driving variables.

We partitioned the net stem CO_2_ flux measured in two Scots pine trees over the growing season of 2021 into stem respiration, stem photosynthesis, and transport of CO_2_ in xylem sap. The continuous, long-term measurements of this study allow new insight into the seasonal dynamics of the different processes regulating stem gas exchange, especially respiration and photosynthesis. Our hypotheses were that: (i) the different stem CO_2_ flux components will have distinct daily patterns, and (ii) as a result of the different seasonality of the main driver of each process, the relative contribution of each to net stem CO_2_ flux will vary with the season.

## Materials and methods

### Study site

Chambers were installed at a height of ~15 m on two mature (61-year-old) Scots pine trees having diameter at breast height (DBH) of 19.70 cm and 22 cm, respectively, in a boreal, evergreen coniferous forest at the SMEAR II station in Hyytiälä (N 61 50.80, E 24 17.70, 180 m.a.s.l.), Finland. The range of measurements utilized in this study included data from June to September 2021. The soil type at the site is glacial till, and the forest floor was dominated by dwarf shrubs and mosses. The long-term annual mean precipitation and the annual mean temperature are 711 mm and 3.5 °C, respectively (Pirinen *et al.*, 2012).

### Measurements

**Fig. 1.**
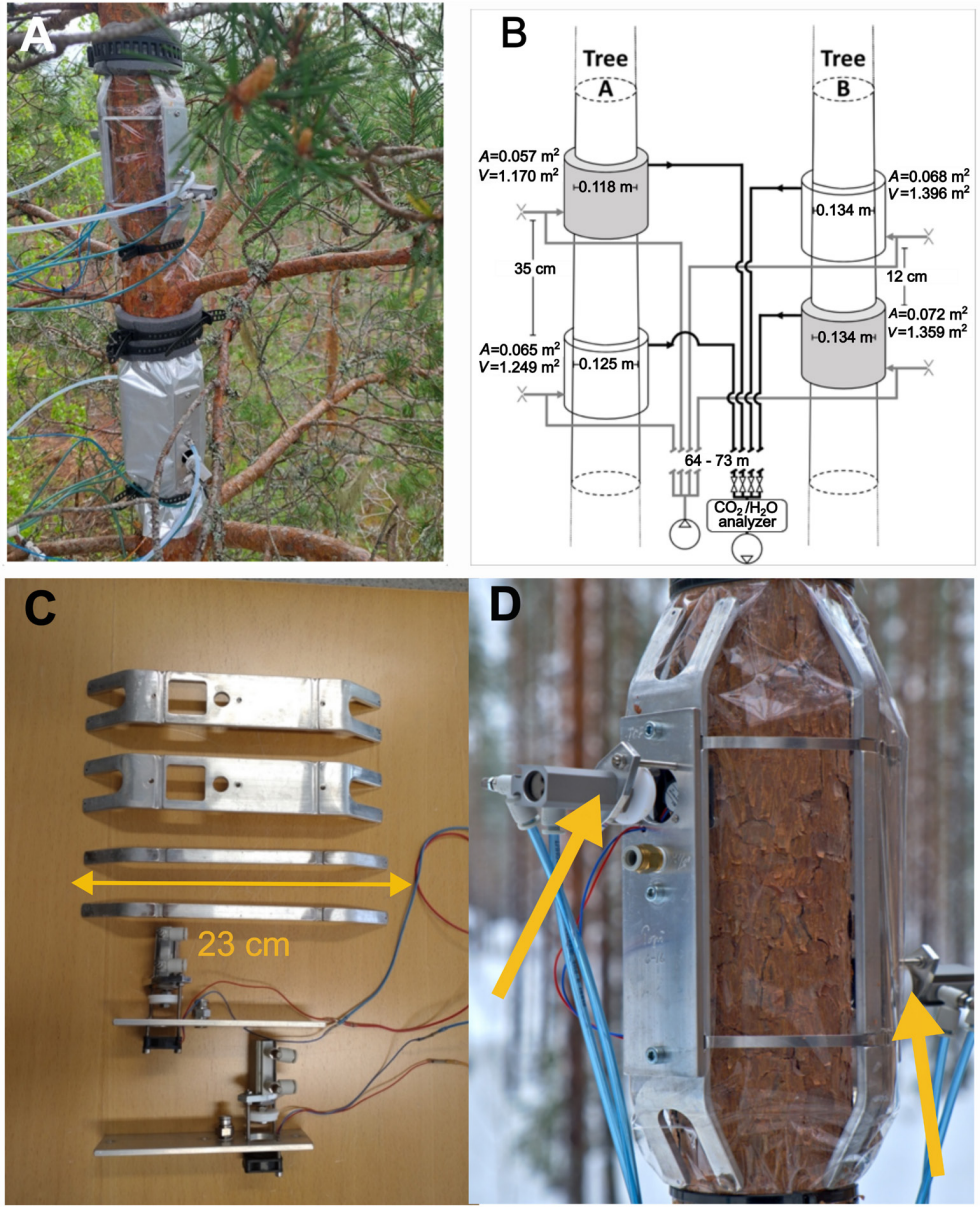
Chamber set-up and CO_2_ efflux measurements. (A) Position of the chambers. (B) Schematic diagram of chamber measurement design. (C) Components of the chamber. (D) Chamber lid positions with one of the lids in open position; arrows indicate the chamber opening positions. The chambers were installed one above the other at a height of ~15 m. The trees are ~2 m apart.

The gas exchange measurement system, consisting of chambers, sampling set-up and gas analyser, allows continuous measurement of stem CO_2_ efflux. The stem chambers were built from two sections of bent aluminum frames with pneumatically operated lids and fans (one lid and fan per section, two sets of lids and fans per chamber), and two sections of smaller aluminum frames keeping the fluorinated ethylene propylene (FEP) foil (chemically inert and solvent resistant to most chemicals) separated from the tree stem (Fig. 1). In the case of dark chambers, the FEP foil was covered with double-layered material consisting of aluminum foil and thin polyvinyl chloride (PVC) film (the outermost surface material). The height of the frames was 23 cm and the maximum gap between the stem and the chamber wall was 23 mm, but considerably less in between the frame components. To ensure free air flow and air mixing inside the chamber, the minimum gap between the stem and chamber wall was 10 mm. The chamber volume was 1.170–1.396 liters, depending on the diameter of stem (Fig. 1).

The chamber set-up is a dynamic flow-through system. The lids and fans allowed the conditions inside the chamber to stay close to ambient between measurement periods, by pushing ambient air through the chamber when the chamber was open; the flow of air was maintained by two fans (Ebmpapst 252 N axial fans, Ebmpapst Inc., Farmington, CT, USA) that are able to maintain free air flow of 0.89 liters min^−1^. The diameter of the lid openings was 23 mm.

The system includes a CO_2_ analyser (LI-840A, LI-COR Inc., Lincoln, NE, USA) that measures the CO_2_ concentrations every 5 s for 100 s closures every 30 min. Constant inflow of ambient air through the chambers is maintained during (flow rate: 1.0–1.2 liters min^−1^, equal the sample flow rate) and between (flow rate 0.5 liters min^−1^) the measurements, and the concentration change is measured.

The chambers were placed one above the other (Fig. 1). The first tree (tree A) had a dark chamber above the transparent chamber; the order was reversed on the second tree (tree B). The chamber dimensions can be seen in Fig. 1. Calculation of gas flux was performed after Kolari *et al.* (2012).

The ancillary variables measured were xylem temperature, relative air humidity, and PAR. Xylem temperature was measured continuously with a PT100 temperature sensor inserted into the xylem surface layer at breast height of the two trees. Relative air humidity and PAR were measured above the canopy from the nearby tower at a height of 35 m. The measurement frequency was 1 min. The relative humidity of the ambient air was measured with Rotronic MP102H RH sensors (Rotronic, Switzerland), and PAR with a Li-190SZ quantum sensor (LI-COR Biosciences, UK). Relative humidity inside the chamber was calculated using relative humidity data of ambient conditions corrected with temperature and pressure data from the chamber during the measurement.

Sap flux density was measured with a constant thermal dissipation probe. Pairs of probes (4 cm in length; 2 mm diameter) were inserted into the xylem beneath the chambers on the north side of the stem with vertical separation of 10 cm between the sensors. The sensor pairs were covered with a reflective aluminum shelter, and the reference condition (i.e. sap flux density ~0) was determined from night-time data as the average of seven consecutive nights (Lu *et al.*, 2004). The temperature difference measurement, logged every minute, was converted to sap flow using the original coefficients of Granier (1987) and sapwood area. In this study, we used the dynamics of the sap flow, rather than absolute values, thus not requiring calibration.

### Theoretical framework and assumptions

**Fig. 2.**
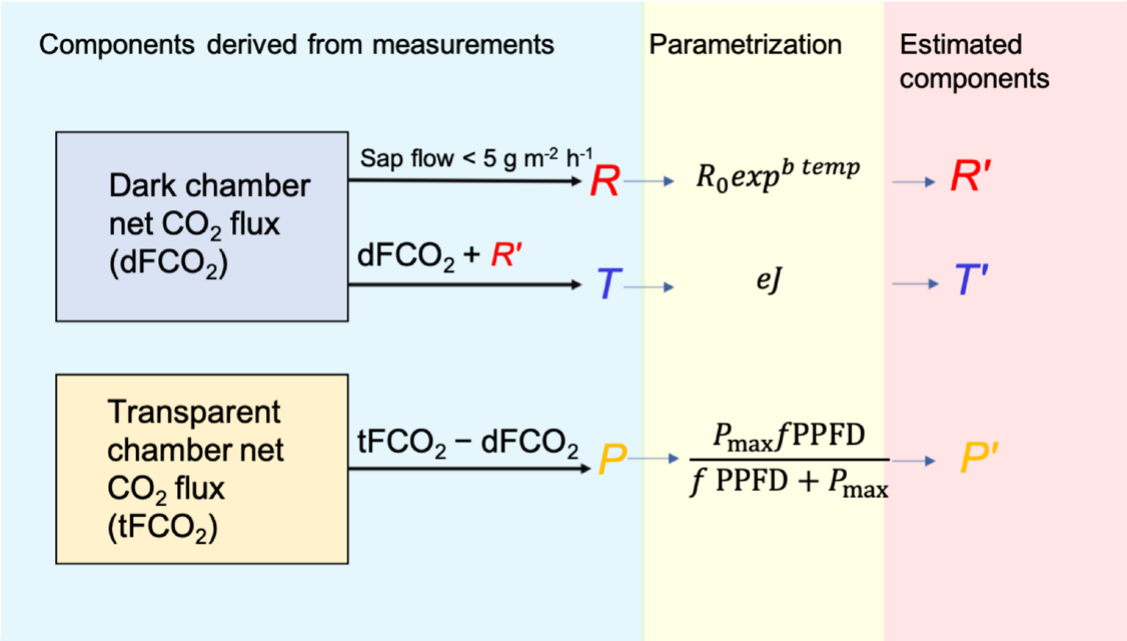
Methodology used to determine the parameters for the components of net stem CO_2_ flux obtained in the partitioning on a monthly basis. *P*, photosynthesis; *R*, respiration; *T*, the axial (vertical) transport of CO_2_. This approach requires data from dark and transparent chambers. The estimation of respiration in the dark chamber was based on conditions of sap flow <5 g m^−2^ h^−1^ (*R*). Next, *Rʹ* was calculated for the entire data range with the derived parameters using xylem temperature (Equation 2). Then, *T*, estimated from the residual of the sum of dFCO_2_ and *Rʹ*, and *P* were calculated and parameterized to model *Tʹ* and *Pʹ*—which describe axial CO_2_ transport in the stem and modelled photosynthesis (Equations 4 and 3, respectively). *b*, the temperature sensitivity of stem respiration; *e*, the slope of the relation between transport and sap flow; dFCO_2_, dark chamber CO_2_ flux; *f*, the slope of photosynthetic light saturation curve at low photosynthetically active radiation (units of μmol CO_2_ μmol^−1^ light quanta); *J*, sap flux density; *P*_max_, light-saturation point of max. photosynthesis; PPFD, photosynthetic photon flux density; *R*_0_, stem respiration at a temperature of 0 °C; tFCO_2_, transparent chamber CO_2_ flux; temp, xylem temperature in the chamber.

The partitioning of stem net CO_2_ flux into different components was performed based on the combined measurements with the dark and transparent chambers, following the mass balance equation (ignoring the storage change of CO_2_):


$$\begin{array}{l} -R+P+T=FCO_{2} \end{array}$$ (1)

where FCO_2_ is the stem net CO_2_ flux, being negative when the flux is directed out of the stem, −*R* is respiratory flux (with adopted sign convention where respiration is always positive), *P* is photosynthesis (always positive), and *T* is transport, representing the contribution of the axial (vertical) advection of CO_2_ to the radial efflux. *T* is equal to the amount of CO_2_ transported axially in xylem sap due to the axial CO_2_ concentration gradient in the stem towards or away from the measurement stem volume. Thus, the transport term, similarly to stem respiration and photosynthesis, is given per unit of stem-section surface area. *T* is estimated as the difference between efflux and respiration in the dark chamber, as will be discussed later (see Fig. 2 and Equation 4). If *P*=0, the mass balance for the chamber requires that *T*≥0, assuming that the sap flow is upwards (positive, or 0). This means that if the amount of CO_2_ respired in the measurement stem volume is less than the amount radially diffused out, then the CO_2_ concentration in the sap has increased across the volume. This leads to a positive axial concentration gradient across the volume and to a positive *T*. Note that *T* should not be confused with the bulk transport of dissolved CO_2_ by the sap flow per unit of cross-sectional area of the stem.

Respiration was parameterized using stem CO_2_ flux data from the dark chamber together with xylem temperature under conditions of no sap flow. For this purpose, the stem temperature inside the chamber was estimated using a differential solution based on temperatures measured in ambient air, the stem outside the chamber, and the air inside the chamber. The difference in the flux between the dark and transparent chambers during daytime was assumed to be photosynthesis. The residual of parameterized respiration and measured CO_2_ efflux from the dark chamber represents the transport-related term. In order to partition the net CO_2_ flux among the three components, the following assumptions were adopted: (i) sap flow is the same at the two positions on the stem where dark and transparent chambers were installed; (ii) stem temperature is the same at the two locations; (iii) there is no acclimation to no-light conditions in the dark chamber; (iv) There are no interactions between photosynthesis and CO_2_ transport (i.e. intercellular CO_2_ concentration); (v) the stem tissue is homogeneous; and (vi) advective transport of CO_2_ is in steady state. Assumption (i) is correct as long as there are no branches between chambers, or when the analysis is based on sap flow dynamic rather that absolute values; the latter is the case here. Assumption (ii) is justified in the fact that the chambers are installed in close proximity and encircle the entire stem, thus averaging over sections of direct and indirect radiation input. Assumption (ii) was introduced to estimate *P* as a simple difference of efflux measured in dark and transparent chambers. Because *T* and *P* occur simultaneously and are prone to cross-correlations in the modeling of the data from the transparent chambers, the assumption allows for a more robust tracing of the *P* and *T* dynamics during the growing season. Assumption (iii) was used to calculate photosynthesis as the difference between the efflux measured in the two types of chambers. We have no means of assessing this assumption. Potential interactions between photosynthesis and CO_2_ transport [assumption (iv)] may affect the estimated *P* and *T* components. We were unable to account for such interactions if present. We assumed that the possible error resulting from these interactions does not significantly affect the final results. The proximity of the chambers and their size allow integration over small scale variability; thus, assumption (v) is likely justified. Assumption (vi) was necessary, as in practice the CO_2_ concentration in stems changes all the time, which makes it difficult to address and partition with averaging time (1 h). Here for transport of mass, steady state means a constant flux.

Stem respiration was assumed to be a function of stem temperature and was described using the classical form (Arrhenius, 1889; Kolari *et al.*, 2014):


$$\begin{array}{l} R^{\prime }=R_{0}exp^{b\times temp} \end{array}$$ (2)

where *Rʹ* (μmol m^−2^ s^−1^) is parametrized stem respiration, *R*_0_ is stem respiration at 0 °C, *b* is the temperature sensitivity, and temp is xylem temperature in the chamber (°C).

The light response curve of stem photosynthesis can be described in the Michaelis–Menten form and its variations (Ye *et al.*, 2012; Lobo *et al.*, 2013; Wei *et al.*, 2017; Li *et al.*, 2019). In this work, a basic formula for the relationship between light and stem photosynthesis was used (Hari and Mäkelä, 2003):


$$\begin{array}{l} P^{\prime }=\frac{P_{max}fPPFD}{fPPFD+P_{max}} \end{array}$$ (3)

where *Pʹ* (μmol m^−2^ s^−1^) is parametrized photosynthesis, PPFD (μmol m^−2^ s^−1^) is photosynthetic photon flux density, *P*_max_ is the light-saturation point or maximum photosynthesis (μmol m^−2^ s^−1^), and *f* is the slope at low PAR (quantum yield).

The effect of transport in xylem sap on local stem CO_2_ efflux (*Tʹ*) was described with a linear relationship (Negishi, 1979) between the measured sap flow and the advective contribution, which was calculated as the residual of parameterized respiration and measured dark-chamber efflux:


$$\begin{array}{l} T^{\prime }=eJ \end{array}$$ (4)

where *Tʹ* (μmol m^−2^ s^−1^—on stem surface) is the axial transport term affecting the CO_2_ efflux, *J* (gH_2_O m^−2^ s^−1^—on sapwood surface) is sap flux density, and *e* is the slope of the linear relationship between *T* and *J*. Note that *e* (μmol m^−2^ s^−1^) represents the axial gradient of the sap CO_2_ concentration over the chamber. Negishi (1979) provided evidence that CO_2_ efflux from the stem was inversely correlated with sap velocity. In a manipulative experiment on excised stems of *Pinus densiflora* trees, he found that CO_2_ efflux was inversely proportional to sap velocity, decreasing by 50% when sap velocity increased from 0 to 0.4 cm min^–1^. In this study, the parameter *e* is fitted at monthly intervals to account for the intra-seasonal variation.

During stem CO_2_ flux data pre-processing, measurements were filtered and subjected to quality control. First, the data were subjected to a technical quality check regarding the system flow rate, pressure and maximum noise in the CO_2_ signal. Measured net CO_2_ flux data (FCO_2_) exceeding the accepted ranges (three standard deviations of the measured dataset) and those when relative humidity >85% were removed to avoid possible condensation in the system. These removed data constituted 20% of available data. Data from the dark and transparent chambers were analysed simultaneously to estimate net CO_2_ flux components. Hereafter, *Rʹ*, *Pʹ*, and *Tʹ* refer to the estimated fluxes based on parametrization of *R*, *P*, and *T*. Note that *T* includes all processes contributing to stem net CO_2_ flux after *R* and *P* are accounted for, thus representing residual flux. For the full representation of the net CO_2_ flux components for day and night, *Rʹ* was used together with measured *P* and *T*. The parameterization of *P* and *T* was used mainly to define the seasonality of the characteristics which describe a given flux. The scheme of CO_2_ flux partitioning into components is presented in Fig. 2. We used the dark chamber data to derive *R* and *T*. Parameterization of *R* was performed using xylem temperature (Equation 1), when sap flux <5 g m^−2^ h^−1^ (corresponding to 5% of the maximum hourly sap flow of the season). Next, *R* was calculated for the entire data including light conditions (*Rʹ*) using coefficients estimated in the previous step and subtracted from the measured FCO_2_ from the dark chamber. This allowed to separate contribution of flux assumed to represent CO_2_ transported in xylem sap (*T*). Measured *P* was derived from the difference between dark chamber CO_2_ flux and transparent chamber CO_2_ flux (tFCO_2_−dFCO_2_). Then, stem CO_2_ assimilation and CO_2_ transport in xylem sap were parameterized (*Pʹ*, *Tʹ*) (Equation 2).

### Statistical analysis

The fittings was performed using ‘nls’ and ‘nlsML’ functions from packages ‘Stats’ and ‘Minipack.lm’ in the R environment (Elzhov *et al.*, 2023). These functions determine the non-linear (weighted) least-squares estimates of the parameters of a non-linear model. Each parameter was estimated with its corresponding standard error, and *t*-test statistics (estimate/standard error) were employed to evaluate the null hypothesis that the model parameters were equal to 0 (H0: θ=0).

## Results

**Fig. 3.**
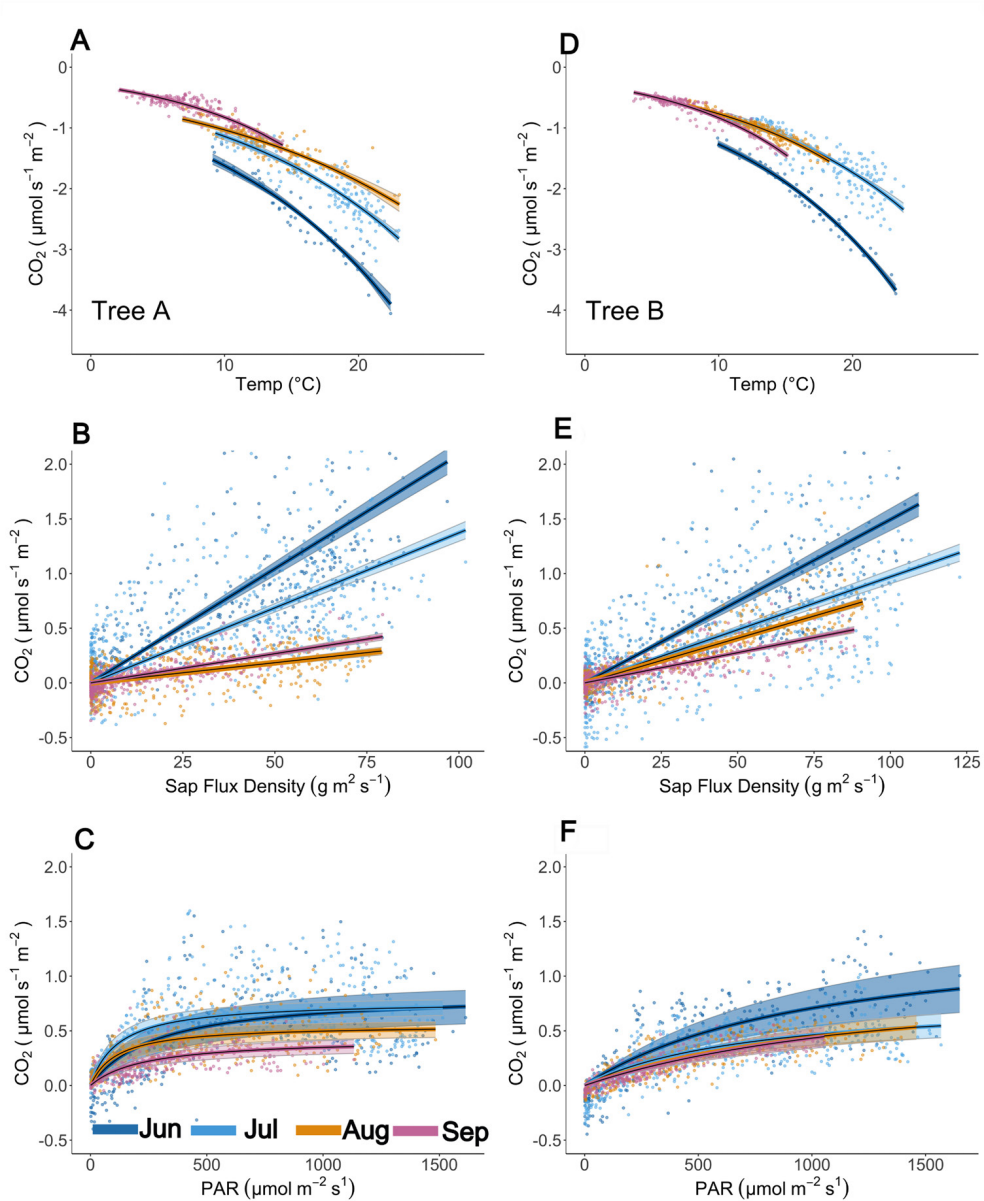
Parameterization of different CO_2_ flux (FCO_2_) components: respiratory flux (−*R*; A, D), transport (*T*; B, E), and photosynthesis (*P*; C, F), for tree A (A–C) and tree B (D–F), for each month from June to September 2021. Data points represent respiration (*R*), transport of CO_2_ in xylem sap (*T*), and photosynthesis (*P*), and the fitted lines represent estimations of these processes due to parametrization, *Rʹ*, *Tʹ*, and *Pʹ*, respectively (Equations 2–4; Fig. 2). Shadowed area corresponds to 95% confidence interval. Note that a negative net flux means efflux from the stem to the atmosphere. For all coefficients, *P*<0.001. PAR, photosynthetically active radiation.

The fits (*P*<0.05) of the three FCO_2_ components (based on Equations 1–3) are presented in Fig. 3. Although a limited number of data points met the conditions of near zero sap flow in summer (June: *n*=84 for tree A and *n*=82 for tree B), stem temperature explained the variation in *R* well. Despite a large scatter, *P* showed a clear response to light in all months. Note that because of the sign convention, a larger net CO_2_ flux means a smaller net efflux indicated by a positive sign (i.e. efflux is always positive). *T* was positively correlated with sap flux density, which means that, on average, sap flow locally decreased the CO_2_ efflux. Moreover, a seasonal trend was observed in the relationship between *T* and sap flow. Residual standard error (RSE) (Supplementary Table S1; Supplementary Fig. S1) was the lowest for the respiration fitting. Fittings for photosynthesis had the highest RSE. For both stem photosynthesis and transport, RSE decreased towards the end of the growing season, with the highest values of RSE in June.

**Fig. 4.**
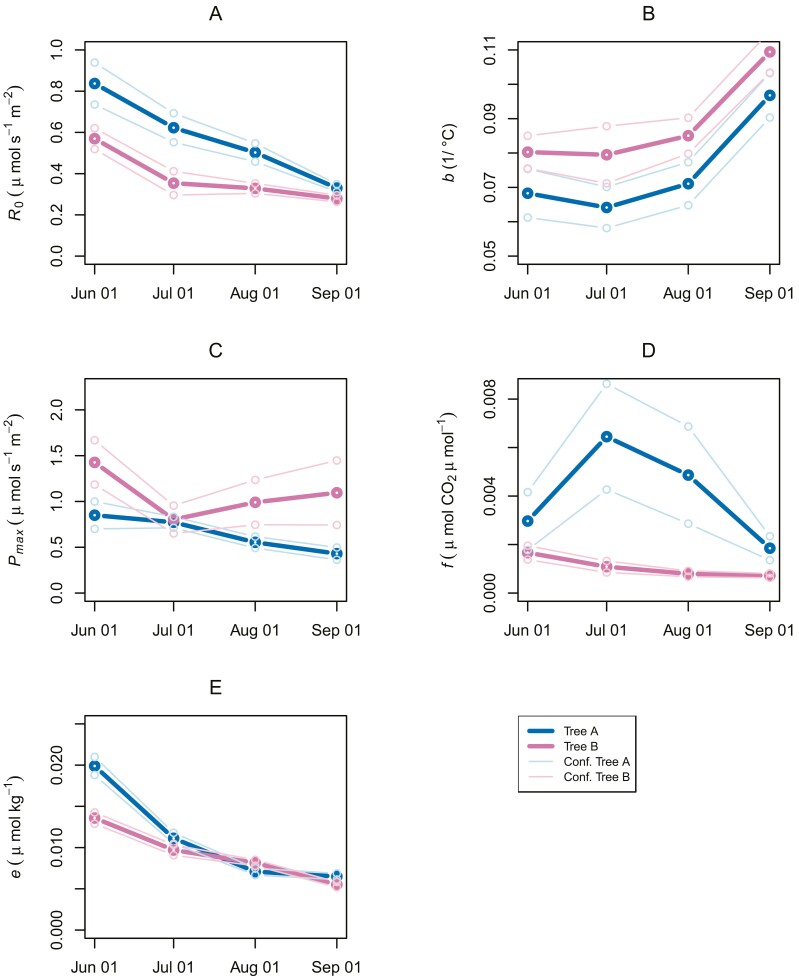
Seasonal courses of derived parameters for tree A (blue) and tree B (red). Light blue and pink lines indicate 95% confidence intervals for tree A and tree B, respectively. *b*, the temperature sensitivity of stem respiration; *e*, the slope of the relation between transport and sap flow; *f*, the slope of photosynthetic light saturation curve at low photosynthetically active radiation (units of μmol CO_2_ μmol^−1^ light quanta); *P*_max_, light-saturation point of photosynthesis; *R*_0_, stem respiration at a temperature of 0 °C.

The seasonal course of the parameters is presented for both trees in Fig. 4. Stem respiration at 0 °C (parameter *R*_0_) decreased seasonally ranging between 0.3 and 0.8 μmol m^−2^ s^−1^. The temperature sensitivity of stem respiration (*b*) increased during the growing season, reaching the highest values in September. The light-saturation point (*P*_max_) of photosynthesis and the initial slope of the light response curve at low PPFD values (*f*) changed over the growing season differently in the two trees, although the former parameter tended to be highest at the beginning of the growing season. The slope of *T* versus sap flux density clearly decreased over the season, indicating a decreasing axial CO_2_ concentration gradient towards the end of the season. For all coefficients, *P*<0.001.

**Fig. 5.**
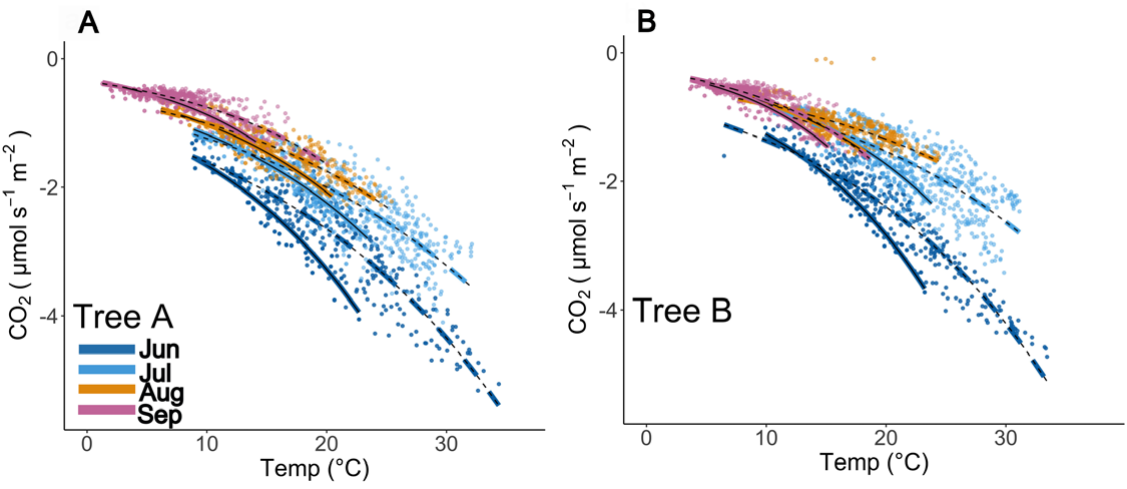
Relationship between estimated CO_2_ flux (sum of −*Rʹ*+*Pʹ*+*Tʹ* estimated based of functions parameterized using 80% of the data) and measured CO_2_ flux (using 20% of the data not used in parameterization) for the transparent chamber of the two trees. Note that a negative net flux means efflux from the stem to the atmosphere. Solid black line represents the relationship as an average for all months. Dashed lines represent relationships in particular months. The thin dashed black shows the 1:1 line.

The methodology used was tested by randomly dividing the data into training and testing groups (8:2). Next, the three parameterized flux components (*Rʹ*, *Pʹ*, *Tʹ*) were summed, which was then compared with the measured flux in the transparent chamber to validate the accuracy of the derived parameters. Figure 5 shows the sum of the three modelled CO_2_ flux components in relation to the measured flux in the transparent chamber, where all three processes influence the flux. To assess the differences in the quality of the parameterization between months, the statistics of the linear function describing the relationship between the measured and the sum of the modelled flux components for the transparent chamber are given in Supplementary Table S2 (see also Supplementary Fig. S1). In total, measured data accounted for 56% and 83% of the variance in the estimated data for tree A and B, respectively. The highest *R*^2^ was calculated for June for tree B.

**Fig. 6.**
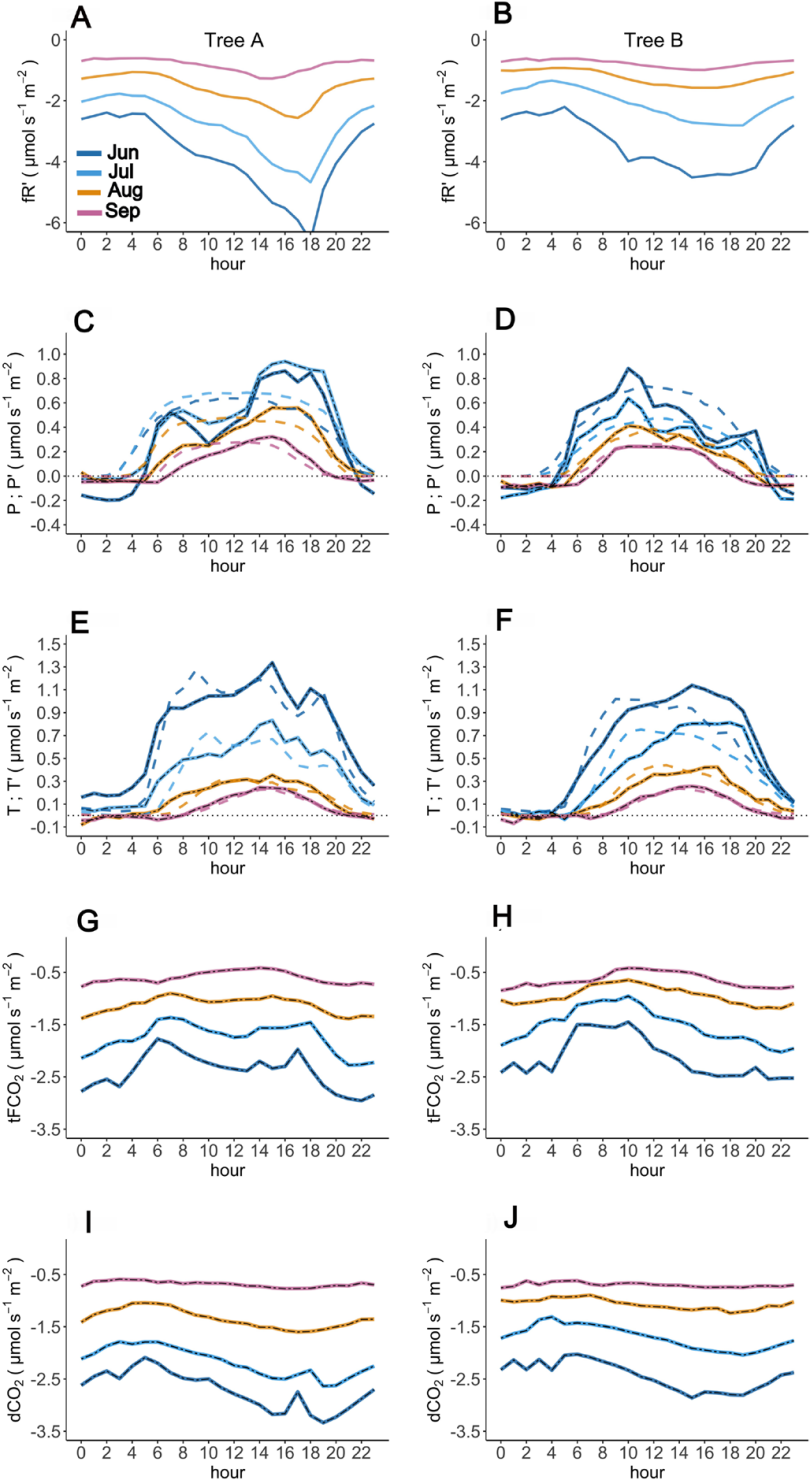
Hourly averages of the parameterized respiratory flux (*fRʹ*; A, B), calculated and parametrized photosynthesis (*P*, *Pʹ*; C, D), calculated and modeled transport (*T*, *Tʹ*; E, F), as well as the measured net flux (FCO_2_) in the transparent (tFCO_2_; G, H) and dark (dFCO_2_; I, J) chambers, for June, July, August, and September 2021.

Diurnal courses and seasonal shares of the different stem net CO_2_ flux components are shown in Fig. 6. In these analyses, we used values derived from measurements for *P* and *T* but parameterized values for *R* (*Rʹ*), because *R* values are limited to conditions with low sap flow (Fig. 2). Seasonally, the highest respiration (lowest respiratory flux −*Rʹ*) occurred in June, decreasing until September (Fig. 6). Naturally, respiration followed the diurnal course of air temperature. The highest respiration occurred in the afternoon, between 16.00 and 18.00 h. The lowest respiration was observed during the coldest hours of the day, around 04.00 h. As the season progressed, respiration decreased and the time of diurnal maximum respiration retreated 1–2 h earlier. For *P*, the highest values occurred in June and July. Diurnally, the time of the maximum photosynthesis differed between the studied trees, reaching highest values at 16.00 h in tree A and at 10.00–11.00 h in tree B. In tree A, the maximum hourly *P* retreated to earlier times of day as the seasons progressed, whereas tree B showed a more stable time of the maximum hourly *P*. *T* was highest during the afternoon hours, mostly between 14.00 h and 16.00 h depending on the season (Fig. 6). In particular, the highest values of axial transport of CO_2_ (*T*) occurred between 08.00 h and 12.00 h, and the daily maximum moved to earlier times of day towards the end of the growing season. In the morning hours (around 02.00–08.00 h), *T* was close to 0 especially in August and September, becoming negative at times, likely owing to the low residual quantities and contributions of other more minor processes.

The lowest values of net CO_2_ flux (highest efflux) in the dark chamber were measured in the evening at 18.00–20.00 h. The minimum and maximum hourly averages of this process were consistent with those observed for respiratory flux, expected given that respiration is the dominant process in the dark chamber. In the transparent chamber, the influence of photosynthesis and CO_2_ transport was apparent, reflected in increasing FCO_2_ (decreasing the efflux) at noon and afternoon. In the transparent chamber, averages of FCO_2_ varied more than in the dark chamber over the day and the season.

**Fig. 7.**
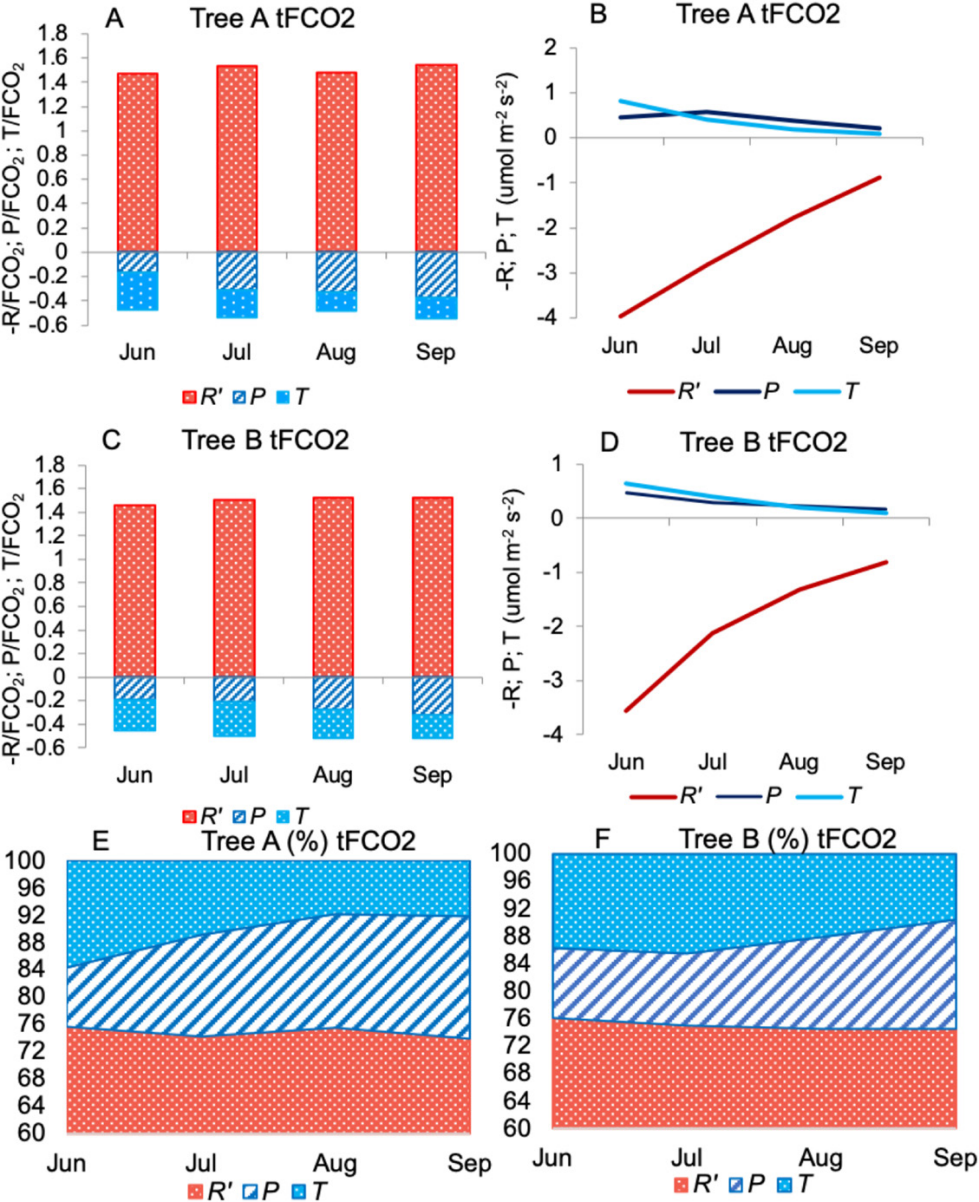
Seasonal ratios of individual processes constituting stem net CO_2_ flux. (A–D) The monthly averages of ratios of individual processes: respiratory flux (−*Rʹ*), photosynthesis (*P*), and transport (*T*) to the net flux in transparent (tFCO_2_) tree A (A) and tree B (C) and the corresponding values (B, D). The ratios in (A, C) are summed to 1 according to Equation 1 in the form of −*Rʹ*/FCO_2_+*P*/FCO_2_+*T*/FCO_2_=1. Note that FCO_2_<0. (E, F) Monthly averages of fractions (%) of individual processes in the transparent chamber CO_2_ flux (tFCO_2_) for tree A (E) and tree B (F). 100% total FCO_2_ is the sum of the absolute values of *Rʹ* (respiration), *P* (photosynthesis), and *T* (transport of CO_2_ in xylem sap).

The monthly average relative contributions to net stem CO_2_ flux of each process (–*Rʹ*, *P*, *T*) are presented in Fig. 7. Although in absolute values stem photosynthesis was highest in June and July (Fig. 6C, D), we observed a clear seasonal increase of the contribution from photosynthesis and decrease of the contribution from CO_2_ transport to the net CO_2_ flux. The share of the three components (*Rʹ*, *P*, *T*) contributing to stem CO_2_ flux was investigated monthly (Supplementary Fig. S1), setting the total FCO_2_, i.e. the sum of the absolute values of *Rʹ*, *P*, and *T*, to 100% (note that we neglect the different signs of the values to compare their relative role). In general, respiration accounted for most of the FCO_2_. The share of photosynthesis and the axial CO_2_ transport in xylem was similar and their importance varied between the trees. The two evident seasonal changes in the proportions were the decrease in the proportion of transport in the CO_2_ flux in favor of photosynthesis and respiration as the growing season progressed, observed clearly in both trees. The relative contribution of photosynthesis increased towards the end of the growing season, reaching 18% and 16% in September for trees A and B, respectively. Respiration made a stable relative contribution to the net CO_2_ flux, varying for both trees between 74% and 76% during the investigated period. The highest *Rʹ* proportion was in September (79% for both trees). The contribution of *T* varied between 8% and 16% for tree A and 10% and 14% for tree B with lowest values in August (tree A) and September (tree B).

## Discussion

The approach of obtaining continuous CO_2_ efflux (−FCO_2_) measurements in dark and transparent chambers on the same tree together with measurements of sap flux, xylem temperature, and PAR allowed us to determine the individual contribution of respiration, photosynthesis, and transport to net stem CO_2_ efflux, as well as the seasonal course of the key parameters describing these three main contributing processes. The approach allows direct estimation of respiration, stem photosynthesis as the difference in CO_2_ efflux between the two chambers, and the contribution of transport or processes correlated with transport.

In this study, stem respiration was the largest component of FCO_2_, contributing 77–79% of the stem net CO_2_ flux. In a previous study of mature (90-year-old) *Pinus sylvestris*, mass balance calculations showed that the major part of the locally respired CO_2_ diffused radially into the atmosphere (Tarvainen *et al.*, 2021), and the respiration represented on average 95% of the observed CO_2_ efflux to the atmosphere. The lower share of respiration found in our study is most likely related to different climate conditions and day length. The measurement of Tarvainen *et al*. (2021) was done in northern Sweden (64°10ʹN, 19°45ʹE, 153 m above sea level) in late summer. This means that stem photosynthesis and transport of CO_2_ in the xylem sap contributing to the net CO_2_ flux were likely lower in their study due to lower evaporative demand and lower light levels. In our study stem respiration reached its highest value in June (6 μmol m^−2^ s^−1^ for tree A), probably due to growth respiration. Indeed, the growth rate of Scots pine in the studied forest has been shown to be highest in June (Chan *et al.*, 2016, 2018). Respiration was lowest in September, ca 1 μmol m^−2^ s^−1^. In August, *Rʹ* reached 4 μmol m^−2^ s^−1^, similar to the August midday rate of *Picea abies* stems in the Moravian–Silesian Beskydy Mts, in a climate classified as subarctic (Köppen climate classification: Dfc) (Kottek *et al.*, 2006; Acosta *et al.*, 2008). The published diurnal maximum stem respiration rate of Scots pine in the boreal zone is 1.5–1.8 μmol m^−2^ s^−1^ in June and July (Zha *et al.*, 2004), whereas that of a subalpine lodgepole pine (*Pinus contorta* ssp. *latifolia*) also located in a climate classified as subarctic ‘Dfc’ ranged between 1.5 and 4.0 μmol m^−2^ s^−1^ depending on development stage and age of the stand (Ryan and Waring, 1992). Indeed, besides temperature, stem respiration is also affected by, for example, age and size of a tree, as well as height of the measurement point. Respiration for hinoki cypress (*Chamaecyparis obtusa*) has been found to increase with tree height (Araki *et al.*, 2010), and was highest above the crown base. For loblolly pine (*Pinus contorta*), respiration at 25 °C ranged from 1.7 µmol m^−2^ s^−1^ in a non-fertilized treatment to 3.3 µmol m^−2^ s^−1^ in a fertilized treatment, which shows that nutrient availability and related growth rate is another important factor affecting in this process (Maier *et al.*, 1998).

Given the temperature sensitivity of *Rʹ*, the highest hourly values of respiration occurred during the warm hours, reaching maximum values in the afternoon (16.00–18.00 h), as has been shown in previous research on Scots pine trees in boreal conditions (Zha *et al.*, 2004). The maximum hourly averages of stem respiration observed here is close to that measured on Scots pine needles during the peak expansion period after bud burst (Zha *et al.*, 2001). Stem respiration dropped to ~1 μmol m^−2^ s^−1^ in September, a value comparable to the needle respiration rate approximately 40 d after bud burst (Zha *et al.* 2001). A previous study found that stem respiration of boreal Scots pine was more sensitive to temperature during the growing season than outside of that period (Zha *et al.*, 2004). In this study, the sensitivity of respiration to temperature increased during the growing season, reaching highest rates in September. This can be explained by higher temperature sensitivity of maintenance respiration compared with that of growth respiration (Penning de Vries *et al.*, 1974; Slot and Kitajima, 2015) as growth has already finished by the start of September. Our results regarding the temperature sensitivity parameter imply that using the partitioning method may allow distinguishing between the periods in which respiration is dominated by growth versus maintenance.

Studies have shown that that stem photosynthesis can contribute a substantial amount to plant net carbon gain (Yiotis *et al.*, 2008) offsetting some of the respiration cost. Stem photosynthesis can amount to 16–60% of leaf photosynthesis in non-succulent plant species (Ávila *et al.*, 2014) and depends on the intensity of photosynthetically active radiation and temperature (Cernusak and Marshall, 2000). Here, the contribution of photosynthesis to the net stem CO_2_ flux reached 11–13%, and the area-specific maximum rate of stem photosynthesis corresponds to ~7% of the maximum leaf-area-specific pine shoot photosynthesis rate measured at the site (Kolari *et al.*, 2014). In holly (*Ilex aquifolium*), net photosynthesis increased with light, with rates close to 1 μmol m^−2^ s^−1^ under PAR of ~100 μmol m^−2^ s^−1^, reaching a maximum of 2 μmol m^−2^ s^−1^ (Pfanz *et al.*, 2002). Similarly, we found that photosynthesis increased with light. The time of maximum values of *P* within a day varied seasonally, and was observed as two peaks, one at 10.00–12.00 h and another at 14.00–16.00 h. This was most likely related to diurnal patterns of shading by branches within the crown and by other crowns (i.e. the local light environment). Seasonally, the hourly average of photosynthetic rate reached 0.8 μmol m^−2^ s^−1^ in June and July when solar angle and PAR are highest. This value is lower than the one reported for European beech (*Fagus sylvatica*) in a typical oceanic climate (Köppen climate classification: Cfb) where maximum values occurred in May just after budburst (ca 3.5 μmol m^−2^ s^−1^) (Berveiller and Damesin, 2008); in June, the values decreased significantly and remained low till winter (less than 0.5 μmol m^−2^ s^−1^). Much higher values were observed in 1-year-old stems of silver birch (*Betula pendula*), where photosynthetic rates reached 4 μmol m^−2^ s^−1^ during midday in August (Wittmann and Pfanz, 2007). Damesin (2003) showed that the photosynthesis of *Fagus sylvatica* stems in a temperate climate was highest at the beginning of the growing season in April, when it accounted for 50% of the monthly respiration. In our study, although photosynthesis reached its highest absolute values at the beginning of the period in June, stem photosynthetic refixation share of the total stem CO_2_ flux was highest in September when photosynthesis comprised 16% and 18% of net CO_2_ flux (as monthly average) and 21% and 24% of monthly respiration for tree A and tree B, respectively; in June the photosynthetic refixation share of respiration was much lower, 11% and 13%, respectively. Cernusak and Marshall (2000) found that maximum observed refixation rate of CO_2_ within a stem of western white pine (*Pinus monticola*) ranged from 1.1 to 2.3 µmol m^−2^ s^−1^, comprising a much higher share, 76 ± 3%, of dark respiration (Cernusak and Marshall, 2000). Warmer climates with extended growing season and high light availability generally promote higher photosynthetic refixation rates.

Based on previous studies, cambial activity and shoot growth begins in the spring prior to bud break, when stem and bark tissues of evergreen trees are the main sink for carbohydrates produced by overwintering needles (Egger *et al.*, 1996; Ivanov *et al.*, 2006). Under non-limiting soil moisture conditions, a combination of high photosynthesis in woody tissues and low tension in the xylem enhances growth respiration (De Roo *et al.*, 2020*a*). Our lower stem photosynthesis relative to respiration in June may be caused by the much larger stem and thicker bark studied relative to the branches used in other studies (Cernusak and Marshall, 2000; Damesin, 2003). Indeed, in aspen (*Populus tremula*), bark photosynthetic capacity increased with decreasing stem diameter and thinning bark as measurement height increased, from 8.5 μmol m^−2^ s^−1^ at breast height to 12.5 μmol m^−2^ s^−1^ closer to the tree top (Kharouk *et al.*, 1995). Even more related, a vertical pattern in the photosynthetic refixation rates was detected in a mature *Pinus sylvestris*, increasing with height as light availability increased and bark type and chlorophyll content adjusted accordingly (Tarvainen *et al.*, 2018). Although these studies show that a large part of respired CO_2_ is refixed locally, the importance of stem photosynthesis for plant function is perhaps greatest under drought conditions. Bark photosynthesis has been shown to prevent leaf fallout, drop in plant water potential, and drop in sap flow during drought, thus positively affecting embolism refilling, transpiration, and leaf photosynthesis (Bloemen *et al.*, 2013, 2016a; De Roo *et al.*, 2020*a*).

Hourly average of parametrized photosynthesis presents some negative *P* values mostly in August and September (Fig. 6C, D). This suggests that the absolute value of the efflux in the transparent chamber was higher (efflux being negative and smaller; Fig. 2) than that in the dark chamber during periods of low light. Possible reasons for this are the temperature difference between the dark and transparent chamber. Also, more non-structural carbohydrates produced under the transparent chamber due to local photosynthesis and higher temperature might lead to higher respiration (Sevanto *et al.*, 2014); higher stem temperature in one chamber would contradict assumption (ii).

Transport-related CO_2_ flux (*T*) contributed 8–16% of the net stem CO_2_ flux (or 10–20% of respiration) with the highest share in June. In the study of McGuire and Teskey (2004), internal transport constituted 55% of the total daily respiration flux in sycamore tree (*Platanus occidentalis*), and 14–15% in sweetgum (*Liquidambar styraciflua*) and American beech (*Fagus grandifolia*) in humid subtropical climate (Köppen climate classification: Cfa) (McGuire and Teskey, 2004). This suggests that the contribution of each flux to net CO_2_ flux varies greatly depending on species and/or environment. In our study, the highest relative contribution of *T* was observed in September. The values of *T* and sap flux were highest in July, coinciding with high values of stem photosynthesis. In the morning hours, *T* was often negative. Fundamentally the negative *T* reflects the non-photosynthetic consumption of CO_2_ in the stem because *T* is determined based on the dark chamber (Fig. 2). The non-photosynthetic process is potentially PEP carboxylase mediated fixation (Hilman *et al.*, 2019; Helm *et al.*, 2023). Beside this, non-stationarities in flow leading to, for example, CO_2_ storage changes may bias the *T* estimates. The approach and data used in this study do not enable determination of the contributions of these processes.

Long-term gas exchange measurements done on mature tree stems in field conditions are challenging and thus rare. Even in this study, possible sources of uncertainty remain: (i) we did not have measurements of local radiation conditions inside the canopy and used above-canopy PAR to explain the variation in bark photosynthesis, and thus not accounting for radiation attenuation and shading by adjacent branches and tree crowns; and (ii) sunlight directly illuminating the chambers can cause unusually high internal temperatures. In addition, although the chambers average the inhomogeneties in xylem, bark thickness, and surface features (Wittmann and Pfanz, 2007; Tarvainen *et al.*, 2018), the location of the chambers may not represent the entire stem.

Our simple set-up allows estimation of the three main processes affecting stem CO_2_ flux—respiration, photosynthesis, and transport in xylem sap. However, recent studies have highlighted the potential role of other processes that might contribute to net CO_2_ efflux, and our approach should be viewed as a base-setting over which some of these factors can be investigated. These include the following: (i) the impact of turgor pressure on cell growth and aerobic mitochondrial respiration, which could partially explain our observation that the net CO_2_ flux at a reference temperature decreased (was more positive) around mid-summer (Tarvainen *et al.*, 2021); (ii) the impact of pH of the xylem water on the dissolved carbon transport (Tarvainen *et al.*, 2023). Tarvainen *et al.* (2023) suggested that *Pinus sylvestris* is one of the species that seems to carry little dissolved carbon in its sap due to low sap pH. This is consistent with an earlier study suggesting that xylem CO_2_ transport is generally limited in conifers (Tarvainen *et al.*, 2021); (iii) the role of PEP carboxylase in reducing diffusive CO_2_ efflux from tree stems (Helm *et al.*, 2023); and (iv) the impact of light-induced axial CO_2_ diffusion along the stem on net CO_2_ flux, reported to be a small but significant factor especially when sap flow rate is low (De Roo *et al.*, 2019, 2020b). Despite the remaining uncertainties, the simple approach coupling a pair of dark and transparent chamber for continuous CO_2_ efflux with sap-flux measurements allowed partitioning stem CO_2_ flux into the three main simultaneous, contributing processes, facilitation the modeling of stem CO_2_ efflux for eco-physiological and biosphere–atmosphere exchange studies.

### Conclusions

Long-term measurements of stem CO_2_ efflux, with a pair of dark and transparent chambers, and sap flux can provide information on seasonal dynamics and drivers of the different processes contributing to stem gas exchange. The results allowed us to conclude that dark and transparent chambers used together allow partitioning of net stem CO_2_ flux to respiration and photosynthesis. Additional sap flux measurements allow us to additionally estimate the effect of CO_2_ transport in xylem sap. The three studied processes contributing to the net stem CO_2_ flux showed distinct seasonal and daily patterns, with varying relative contribution over the growing season, consistent with our hypotheses. Stem photosynthesis was highest in June and July; however, the share of photosynthesis increased towards the end of the growing season from June to September, mainly due to higher reduction in respiration and CO_2_ transport in sap in relation to stem photosynthesis. Stem respiration at a reference temperature decreased seasonally. The temperature sensitivity of stem respiration (*Q*_10_) increased during the growing season and was highest in September, when maintenance respiration dominates in this biome. The light-saturation point of photosynthesis (quantum yield) decreased from June to September. The initial slope of the light response curve at low PAR values declined seasonally.

## Supplementary data

The following supplementary data are available at *JXB* online.

Fig. S1. Monthly averages of fractions (%) of individual processes in the dark chamber CO_2_ flux.

Fig. S2. Relationship between FCO_2_ (CO_2_ flux) flux xylem temperature as well as relationship between respiration (−*R*) and xylem temperature.

Table S1. Residual Standard Error (RSE) and number of observations used (*n*) for fitting the variables (*R*, *P*, *T*).

Table S2. Comparison of modelled versus measured CO_2_ flux in the transparent chambers of the two trees—slope and intercept of the linear relationship, *R*^2^, and residual standard error.

erae242_suppl_Supplementary_Figures_S1-S2_Tables_S1-S2

## Data Availability

Data will be made available on request.
