# Supplementary material for: Partitioning seasonal stem carbon dioxide efflux into stem respiration, bark photosynthesis, and transport-related flux in Scots pine
Source: J Exp Bot. 2024 May 23;75(16):4944–59. doi: 10.1093/jxb/erae242 (PMC11350082; doi:10.1093/jxb/erae242)
Supplement: erae242_suppl_Supplementary_Figures_S1-S2_Tables_S1-S2 [file erae242_suppl_supplementary_figures_s1-s2_tables_s1-s2.pdf]

Supplementary material

Partitioning seasonal stem carbon dioxide efflux into stem respiration, bark photosynthesis and transport-related flux

Paulina Dukat [1,2], Teemu Hölttä [1], Ram Oren [1,3] Yann Salmon [1,4], Marek Urbaniak [2], Timo Vesala [1,4], Juho Aalto [1], Anna Lintunen [1,4]

[1] Institute for Atmospheric and Earth System Research/Forest Sciences, Faculty of Agriculture and Forestry, University of Helsinki

[2] Laboratory of Meteorology, Department of Construction and Geoengineering, Faculty of Environmental Engineering and Mechanical Engineering, Poznan University of Life Sciences, Piątkowska 94, 60-649 Poznań, Poland

[3] Nicholas School of the Environment & Pratt School of Engineering, Duke University, Durham, North Carolina

[4] Institute for Atmospheric and Earth System Research/Physics, Faculty of Science, University of Helsinki

Table S1. Residual Standard Error (RSE) and number of observations used (n) for fitting the variables (R, P, T) for Tree A and Tree B. The significance of all obtained fitting parameters < 0.001.

		RSE/ Tree		n	
**Month**	**Variable**	**T_A**	**T_B**	**T_A**	**T_B**
**June**	**R'**	0.21	0.12	84	82
	**P'**	0.32	0.23	325	353
	**T'**	0.47	0.51	286	309
**July**	**R'**	0.24	0.24	156	159
	**P'**	0.27	0.17	573	596
	**T'**	0.34	0.41	437	450
**August**	**R'**	0.15	0.07	140	153
	**P'**	0.20	0.10	398	447
	**T'**	0.14	0.14	296	318
**September**	**R'**	0.09	0.08	200	178
	**P'**	0.11	0.07	468	318
	**T'**	0.09	0.10	288	294

Table S2. Comparison of modelled vs. measured CO2 flux in the transparent chambers of the two trees. The modelled CO2 flux is the sum of the modelled respiration (R'), photosynthesis (P') and

transport component (T') according to eq. 1. Slope and intercept of the linear relationship, R-squared (R2), and residual standard error (RSE) are presented for each month separately, as well as for the entire observation period.

	slope		*intercept*		R^2		*RSE*	
	Tree A	*Tree B*	*Tree A*	*Tree B*	*Tree A*	*Tree B*	*Tree A*	*Tree B*
June	0.99**	1.25**	-0.40*	0.58**	0.35	0.91	1.23	0.39
July	0.76**	0.88**	-0.68**	-0.15**	0.24	0.70	0.83	0.38
August	1.65**	0.94**	-0.57**	-0.02·	0.11	0.78	0.58	0.15
September	0.67**	0.83**	-0.27**	-0.07**	0.37	0.83	0.19	0.11
Total	1.03**	1.01**	-0.18**	0.03^	0.56	0.83	0.78	0.37

Signif. codes: p value: 0 - 0.001'**' 0.0011-0.01 '*' 0.011-0.05 '^' 0.051 -0.1 '·' 0.11< '·'

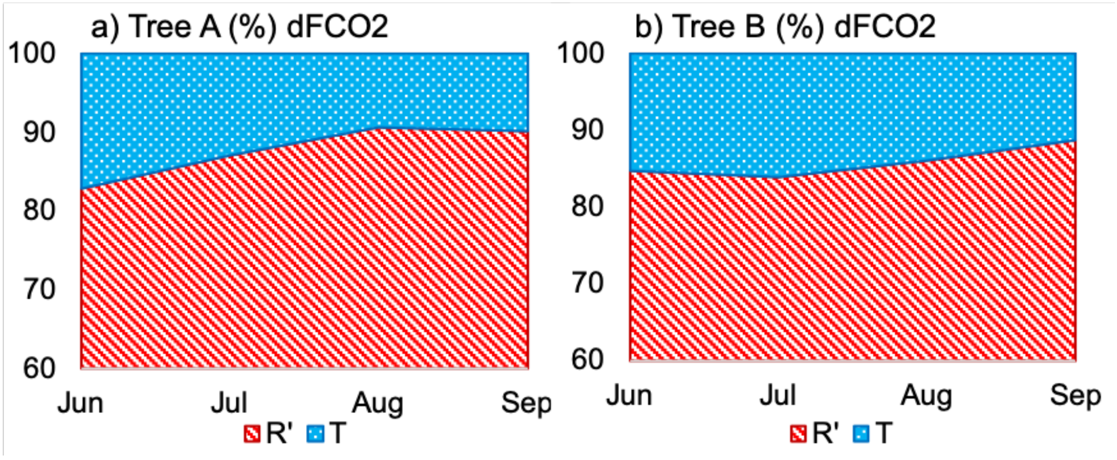

Figure S1. Monthly averages of fractions (%) of individual processes in the dark chamber CO_2 flux ($dFCO_2$) for tree A (a) and tree B (b). 100% total FCO_2 is the sum of the absolute values of R' (respiration) and T (transport of CO_2 in xylem sap).

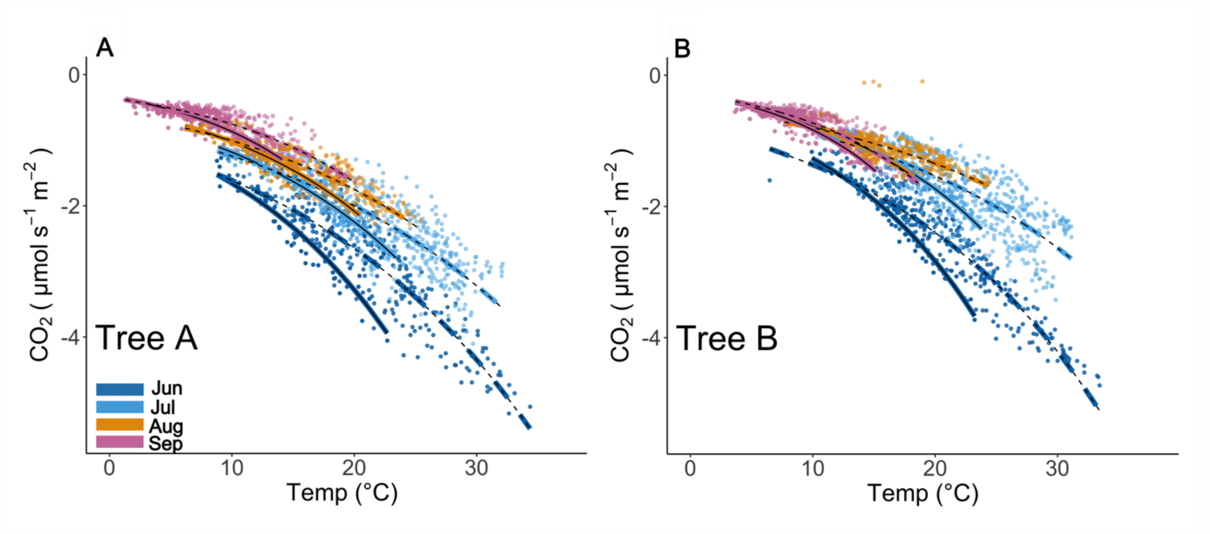

Figure S2. Relationship between FCO_2 (CO_2 flux) flux and xylem temperature as well as relationship between respiration (-R) and xylem temperature; tree A (a) and B (b), for each month from June to September 2021. Solid fitted lines represent R', dashed lines represent FCO_2. R' was parametrized for the conditions when sap flow < 5 g m^{-2} s^{-1}.
